# Clinical Outcomes of Ultrasound-Guided Stellate Ganglion Block in Highly Treatment-Resistant Chronic Migraine: A 6-Month Prospective Single-Group Interventional Study

**DOI:** 10.3390/medicina62091784

**Published:** 2026-09-16

**Authors:** Sukriye Dadali, Emel Basar, Ulku Sabuncu, Gulcin Babaoglu, Ali Costu, Erkan Yavuz Akcaboy

**Affiliations:** 1 Department of Pain Medicine, Ankara Bilkent City Hospital, Ankara 06800, Turkey; sabuncuulku@gmail.com (U.S.); gulcinpektasli@gmail.com (G.B.); alicostu@gmail.com (A.C.); akcaboyyavuz@gmail.com (E.Y.A.); 2 Department of Pain Medicine, Giresun Training and Research Hospital, Giresun 28100, Turkey; dr_emel86@hotmail.com

**Keywords:** migraine disorders, chronic disease, nerve block, stellate ganglion, ultrasonography, treatment-resistant

## Abstract

*Background and Objectives*: Chronic migraine (CM) remains a major cause of neurological disability, and a subset of patients remains highly treatment-resistant despite previous preventive and interventional treatments. Stellate ganglion block (SGB) has been proposed as a sympathetic neuromodulatory intervention, but evidence in this population remains limited. This study evaluated 6-month clinical outcomes and tolerability after a structured ultrasound-guided SGB protocol. *Materials and Methods*: This prospective, single-center, uncontrolled, single-group interventional study enrolled 42 adults with CM who had experienced insufficient benefit from prior preventive migraine treatment and previous interventional headache procedures. After an initial ultrasound-guided SGB to assess anatomical suitability and procedural tolerability, all participants proceeded to four additional weekly ultrasound-guided SGB sessions using 5 mL of 1% lidocaine, for five protocol-defined sessions in total. The primary outcome was the change in monthly headache days (MHD) from baseline to 6 months. *Results*: All 42 participants (mean age: 44.4 ± 7.4 years) completed the 6-month follow-up. Median MHD decreased from 29.0 days at baseline to 6.0 days at 6 months (Kendall’s W = 0.643, *p* < 0.001), corresponding to a median within-participant reduction of 17.0 days (BCa 95% CI: 13.0–19.0). A ≥50% reduction in MHD was achieved by 73.8% of participants (95% CI: 58.9–84.7) at 6 months, whereas 14.3% (95% CI: 6.7–27.8) achieved a ≥50% reduction in mean pain intensity. HIT-6 and MIDAS scores also improved significantly (*p* < 0.001). Six participants (14.3%) reported mild, self-limited adverse effects; no serious adverse events were documented. *Conclusions*: Ultrasound-guided SGB was associated with sustained reductions in headache frequency, analgesic use, and migraine-related disability over 6 months. Because this was an uncontrolled single-group study, the findings should be considered preliminary and hypothesis-generating. The frequency-dominant pattern of improvement and favorable tolerability support further controlled evaluation of SGB as a potential adjunctive neuromodulatory strategy in highly treatment-resistant CM.

## 1. Introduction

Migraine is a highly prevalent and disabling neurological disorder and remains one of the leading causes of years lived with disability worldwide. Chronic migraine represents the most severe form of the disease and is defined according to the International Classification of Headache Disorders, 3rd edition (ICHD-3) as headache occurring on ≥15 days per month for more than three months, of which ≥ 8 days fulfill migraine criteria [1]. Chronic migraine is associated with substantial functional impairment, psychiatric comorbidities, increased healthcare utilization, and reduced quality of life [2].

Despite the availability of diverse preventive therapies—ranging from conventional pharmacological classes such as beta-blockers, anticonvulsants, and antidepressants to advanced options like onabotulinumtoxinA—a considerable proportion of patients continue to experience frequent and disabling attacks. The European Headache Federation (EHF) proposed a clinical distinction between resistant and refractory migraine, defining resistant migraine as failure of at least three classes of preventive therapies administered at adequate dose and duration, while refractory migraine represents a more severe phenotype characterized by ongoing disability despite extensive therapeutic attempts [3]. Real-world data, including findings from the REFINE study, further demonstrate the substantial burden and comorbidity profile of this population [4,5].

However, in routine clinical practice, a substantial subset of patients falls within a highly treatment-resistant spectrum, having failed multiple pharmacological and interventional treatment lines. These individuals often experience persistent disability despite established preventive therapies and prior interventional pain procedures. This population therefore represents a major unmet therapeutic need and a substantial challenge in specialized headache care.

Peripheral nerve blocks are increasingly used as interventional options in chronic migraine care. Recent real-world evidence indicates that repeated combined greater occipital and supraorbital nerve blocks may reduce headache frequency, pain intensity, analgesic consumption, and migraine-related disability, supporting their potential role within multimodal treatment pathways [6].

Beyond the traditional trigeminovascular framework, migraine is increasingly understood as a disorder involving interactions among central nociceptive networks and autonomic regulatory systems [7]. Functional neuroimaging studies have demonstrated dynamic alterations in hypothalamic and brainstem connectivity across the migraine cycle, supporting the concept of migraine as a disorder of network dysregulation rather than a purely vascular phenomenon [7,8]. Clinical investigations have also documented autonomic dysfunction in migraine populations, including altered cardiovascular autonomic responses [9,10].

Recurrent nociceptive drive within the trigeminovascular system has been strongly associated with the development of central sensitization, a key mechanism implicated in migraine chronification [11]. Furthermore, associations between cranial autonomic symptoms and markers of central sensitization have been demonstrated in prospective clinical studies, supporting the concept that autonomic dysregulation and sensitization processes interact in the evolution of chronic migraine [12,13].

Modulation of the cervical sympathetic chain via stellate ganglion block (SGB) targets pre- and postganglionic sympathetic fibers and may influence cranial vascular regulation through attenuation of sympathetic outflow [14,15]. Magnetic resonance angiography in healthy volunteers has demonstrated that SGB predominantly induces changes in ipsilateral extracranial arterial signal intensity and vessel diameter [16]. Similarly, magnetic resonance imaging in patients with acute or chronic pain has shown increased blood flow velocity in the ipsilateral common carotid artery without significant alteration of the vertebral artery, suggesting that the hemodynamic effects of SGB are primarily confined to the extracranial vascular territory [17]. Whether such physiological effects translate into clinically meaningful modulation of migraine-related nociceptive processing, however, remains to be elucidated.

The therapeutic rationale for stellate ganglion block (SGB) in chronic migraine is based on modulation of cervical sympathetic activity, which may influence afferent nociceptive processing and central sensitization [11,13,18]. Interruption of sympathetically maintained signaling has therefore been proposed as a potential mechanism through which SGB could modulate the neuro-autonomic axis [9,15].

Although stellate ganglion block (SGB) is traditionally employed in sympathetically mediated pain conditions, emerging clinical observations suggest that it may provide benefit in selected migraine populations [14,19,20]. Nevertheless, high-quality randomized controlled data specifically addressing outcomes in highly treatment-resistant chronic migraine remain limited.

Therefore, the present study aimed to evaluate 6-month clinical outcomes and tolerability following a structured ultrasound-guided SGB protocol in patients with a highly treatment-resistant chronic migraine phenotype treated in a tertiary pain clinic.

## 2. Materials and Methods

### 2.1. Study Design and Ethical Approval

This was a prospective, single-center, uncontrolled, single-group interventional study conducted at the tertiary pain management clinic of Ankara Bilkent City Hospital between March 2024 and December 2025. The original study protocol received institutional ethics approval before enrollment and intervention on 6 March 2024 (TABED 2-24-33) and initially specified follow-up through 3 months. The study was retrospectively registered at ClinicalTrials.gov (NCT06891118); the initial registration was submitted on 6 March 2025 and first posted on 24 March 2025. During ongoing follow-up and before statistical analysis, a formal protocol amendment was submitted on 30 October 2025 to extend follow-up from 3 to 6 months and to define the final assessment time points as months 1, 3, and 6. The amendment was approved by the institutional ethics committee on 26 November 2025 (TABED 2-25-1653). The ClinicalTrials.gov record was subsequently updated and is now publicly available, reflecting the approved 6-month follow-up and the primary outcome, defined as change in MHD from baseline to 6 months. All participants provided written informed consent before enrollment. The original registry listed VAS, HIT, MIDAS, and GPE as primary outcomes over a 3-month follow-up period. With the extension of follow-up to 6 months, change in MHD from baseline to 6 months was specified as the primary endpoint to provide a clinically relevant measure of headache frequency over the extended follow-up period. No interim statistical analysis or review of outcome data was performed before the amendment was proposed.

### 2.2. Participants

**Figure 1:**
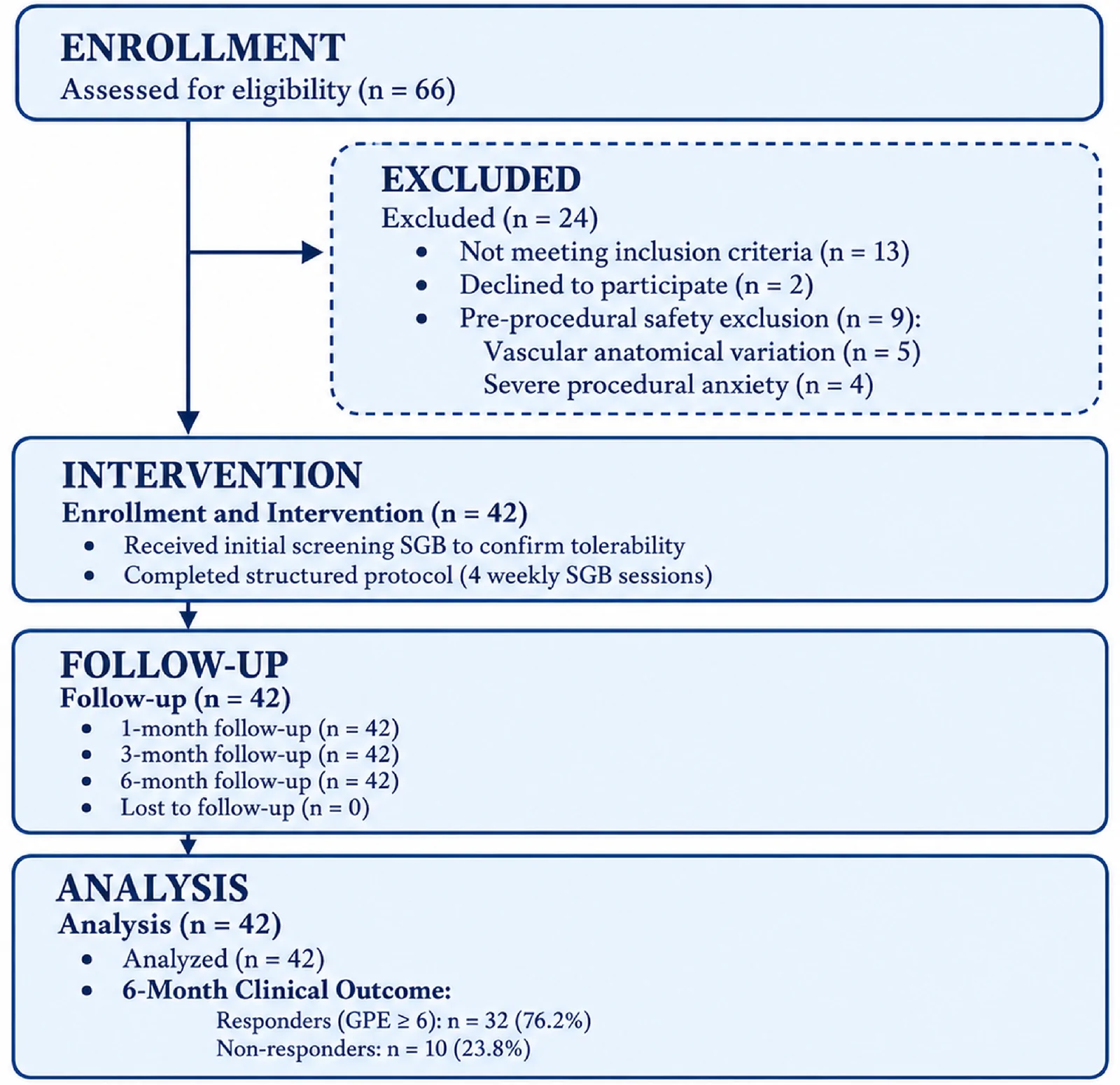
Flow diagram of participant screening, enrollment, intervention, follow-up, and analysis. SGB, stellate ganglion block; GPE, Global Perceived Effect.

Eligible participants were adults aged 18–65 years diagnosed with chronic migraine according to the International Classification of Headache Disorders, 3rd edition (ICHD-3). None of the participants had a history of calcitonin gene-related peptide (CGRP)-targeted therapy, as clinical accessibility was significantly constrained during the study period. During the recruitment period, potentially eligible patients with chronic migraine who were followed at or presented to the pain clinic were screened consecutively for study eligibility (Figure 1).

### 2.3. Definition of Highly Treatment-Resistant Chronic Migraine

Participants had previously received preventive migraine treatment with insufficient clinical benefit and had also undergone at least one interventional headache treatment, performed at least 3 months before enrollment, without satisfactory or sustained improvement. Previous interventional treatments included greater occipital nerve blocks, pulsed radiofrequency procedures, and sphenopalatine ganglion interventions. The cohort included patients with prior onabotulinumtoxinA exposure, while access to CGRP-targeted preventive therapies was substantially constrained during the study period. Preventive treatment histories were heterogeneous; failure of a prespecified number of preventive drug classes was not a formal eligibility criterion.

Based on the substantial baseline headache burden, previous preventive treatment attempts, and extensive exposure to interventional headache therapies, the cohort was operationally described as having highly treatment-resistant chronic migraine. This term is used as a descriptive clinical characterization informed by contemporary EHF concepts [3], not as a separate formal EHF diagnostic category or as confirmation that every participant fulfilled all formal criteria for resistant or refractory migraine.

### 2.4. Exclusion Criteria

Patients were excluded from the study if they met any of the following criteria: history of a primary headache disorder other than migraine or a secondary headache disorder; migraine-related interventional treatment, botulinum toxin A treatment, or nonpharmacological headache treatment within the previous 3 months; infection at the planned injection site; pregnancy or suspected pregnancy; known allergy to local anesthetic agents; history of malignancy or cranial/cervical surgery; bleeding or coagulation disorder or current oral anticoagulant therapy; comorbid conditions that could contribute to headache, such as uncontrolled hypertension or intracranial lesions; conditions that could impair adherence to treatment or follow-up, such as significant psychiatric disease or dementia; anatomical constraints preventing a safe needle trajectory; or unwillingness to undergo the planned intervention or continue the study protocol.

Preventive migraine medications that had been used for more than 3 months before enrollment could be continued during the study provided that the dose remained unchanged. Participants could use their established acute migraine medication as needed. During the SGB treatment and follow-up period, initiation of a new or different migraine preventive or acute medication regimen, antidepressant or sleep-regulating treatment, additional interventional pain procedure, acupuncture, or physical therapy was not permitted.

### 2.5. Stellate Ganglion Block Protocol

All potential candidates underwent a pre-procedural ultrasound assessment to confirm that a safe needle trajectory was feasible. Candidates with significant vascular anatomical variations precluding safe access or with severe pre-procedural anxiety were excluded before injection. Eligible participants then underwent an initial ultrasound-guided SGB to assess procedural tolerability and anatomical suitability under actual treatment conditions. Severe procedural anxiety was determined based on patient-reported symptoms and clinical observation during procedural preparation or needle placement, without the use of a standardized anxiety scale. It was characterized by marked anxiety or fear with observable manifestations such as pronounced trembling, motor restlessness, or avoidance behavior, leading to withdrawal from the procedure or precluding its safe initiation or continuation despite procedural explanation and reassurance.

The initial SGB was not used as a diagnostic peripheral nerve block to select treatment responders and no prespecified quantitative magnitude of early clinical improvement was required for continuation. The protocol allowed participants who perceived no benefit and did not wish to proceed to discontinue after the initial SGB; in practice, no participant elected to discontinue at this stage. All 42 participants proceeded to four additional weekly SGB sessions, resulting in five protocol-defined SGB sessions per participant (210 scheduled sessions across the cohort). Follow-up assessments at 1, 3, and 6 months were referenced to completion of the full five-session SGB course, rather than to the initial SGB.

### 2.6. Intervention: Stellate Ganglion Block

Prior to the intervention, routine laboratory tests were conducted, and all procedures were performed under sterile conditions with intravenous access and continuous hemodynamic monitoring. All SGBs were performed by an experienced pain specialist using a standardized, ultrasound-guided technique. Patients were placed in the supine position with slight cervical extension. A high-frequency linear transducer (10–15 MHz) (Aplio 500; Toshiba Medical, Tokyo, Japan) was used to identify the C6 transverse process, the carotid artery, the longus colli muscle (LCM), and the prevertebral fascia.

**Figure 2:**
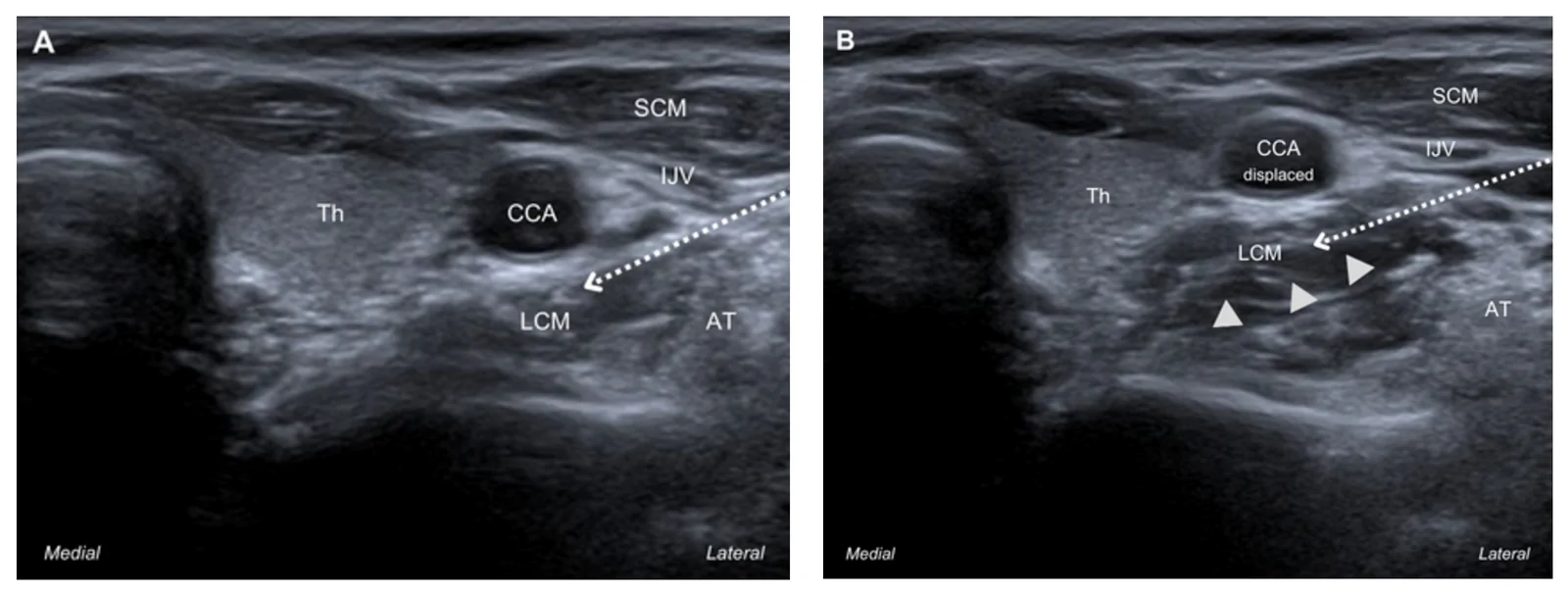
Ultrasound-guided stellate ganglion block technique. (**A**) Baseline axial ultrasound image at the C6 level showing the relationship between the prevertebral structures. The common carotid artery (CCA) is shown in a neutral position, partially obscuring the lateral aspect of the longus colli muscle (LCM). The dashed arrow indicates the intended in-plane needle trajectory toward the prevertebral fascia, directly anterior to the LCM. (**B**) Intra-injection image showing local anesthetic spread beneath the prevertebral fascia (white arrowheads), with secondary ventral and slight medial displacement of the common carotid artery (labeled ‘CCA displaced’), consistent with subfascial drug deposition and compartmental expansion. Abbreviations: SCM, sternocleidomastoid muscle; IJV, internal jugular vein; CCA, common carotid artery; LCM, longus colli muscle; AT, anterior tubercle of C6; Th, thyroid gland. Image orientation is indicated as medial and lateral.

Under real-time sonographic guidance, a 22-gauge, 90-mm needle (Egemen; Izmir, Turkey) was advanced using an in-plane approach from a lateral-to-medial direction targeting the prevertebral fascia, directly anterior to the LCM at the C6 level (Figure 2A). After negative aspiration, needle placement was confirmed with 0.5 mL of normal saline. Subsequently, 5 mL of 1% lidocaine hydrochloride (Aritmal 2% ampule, diluted; Osel Pharmaceutical Industry, Istanbul, Turkey) was slowly injected. During the administration, the increasing volume of the local anesthetic beneath the prevertebral fascia resulted in a ventral (anterior) and slight medial displacement of the common carotid artery (CCA), confirming successful subfascial drug deposition and compartmental expansion (Figure 2B).

Successful sympathetic blockade was clinically confirmed by the presence of Horner’s syndrome (miosis, ptosis, and anhidrosis) within 15 min.

The side of application was determined by headache lateralization; patients with unilateral predominance received ipsilateral SGB, while those with bilateral distribution underwent sequential bilateral blocks with a minimum of a 60-min interval between sides. During this period, participants were monitored via continuous pulse oximetry and clinical assessment for signs of recurrent laryngeal or phrenic nerve involvement to ensure safety before proceeding to the contralateral side.

### 2.7. Outcome Measures

#### 2.7.1. Primary Outcome

The primary endpoint was the change in monthly headache days (MHD) from baseline to 6 months. MHD was also assessed at months 1 and 3 to characterize the longitudinal trajectory. A headache day was defined as a calendar day on which headache lasted continuously for at least 4 h and reached at least moderate intensity, or on which acute medication was used to treat headache regardless of headache duration or intensity. A ≥50% reduction in MHD from baseline was defined as a clinically meaningful response, and responder rates were calculated at each follow-up time point.

#### 2.7.2. Secondary Outcomes

Secondary outcomes included changes in mean and peak headache pain intensity measured using a 0–10 Visual Analog Scale (VAS), monthly severe headache days, attack duration, monthly analgesic-use days, headache-related disability, and patient-perceived improvement. A severe headache day was defined as a headache day with a VAS score ≥ 5. Attack duration was recorded in hours. Analgesic-use days were defined as calendar days on which analgesic medication was used for headache relief. Disability was assessed using the 6-item Headache Impact Test (HIT-6) and the Migraine Disability Assessment (MIDAS) questionnaire [21,22]. Responder analyses evaluated the proportions of participants achieving ≥ 30% and ≥50% reductions in mean pain intensity. Patient-perceived improvement was recorded using a 7-point Global Perceived Effect (GPE) scale [23], ranging from 1 (very much worsened) to 7 (very much improved); clinically meaningful improvement was defined as GPE ≥ 6. MHD, severe headache days, attack duration, pain intensity, analgesic use, and HIT-6 were assessed at baseline and at months 1, 3, and 6; MIDAS was assessed at baseline and at months 3 and 6; and GPE was assessed at months 1, 3, and 6.

Participants were instructed to complete study-specific monthly headache follow-up forms throughout the study and to bring these records to scheduled visits. Study physicians completed clinical follow-up forms using the available headache records together with structured patient-reported information obtained at each visit. Although participants were encouraged to maintain prospective headache records throughout follow-up, completed forms were not available at every visit for every participant; when a form was unavailable, the corresponding follow-up data were obtained through structured patient recall and recorded on the study follow-up form.

### 2.8. Use of Generative Artificial Intelligence

Generative artificial intelligence was used to assist with manuscript drafting, language editing, structural refinement, and journal-specific formatting. It was not used to generate or modify study data, perform statistical analyses, or determine the interpretation of the results. All AI-assisted content was critically reviewed, verified, and edited by the authors, who take full responsibility for the final manuscript.

### 2.9. Statistical Analysis

Sample size estimation was performed a priori, before participant enrollment, using G*Power (version 3.1.9.7) with a two-tailed paired-samples *t*-test. The effect size was derived from the MHD data reported by Yu et al. [20] (23.1 ± 5.5 at baseline vs. 10.9 ± 7.1 at Month 1). Assuming a within-participant correlation of 0.50, Cohen’s dz was 1.891, yielding a minimum sample size of 6 participants at α = 0.05 and 95% power. This minimum was not used as a recruitment stopping criterion; eligible patients meeting the prespecified criteria continued to be enrolled during the study period, resulting in a final sample of 42 participants.

Normality of numerical variables was assessed using the Shapiro–Wilk test. Normally distributed continuous variables are presented as mean ± standard deviation, whereas non-normally distributed continuous variables are presented as median (interquartile range [IQR], 25th–75th percentiles); categorical variables are presented as frequencies and percentages. Longitudinal changes in repeated continuous outcomes were analyzed using the Friedman test, with Kendall’s W reported as an effect-size measure. Post hoc pairwise comparisons with baseline were adjusted using the Bonferroni correction. For the primary endpoint, the within-participant change in MHD from baseline to Month 6 was calculated as baseline minus Month 6 MHD, such that positive values indicated a reduction in headache days. Given the non-normal distribution of the data, the primary effect estimate was expressed as the median paired change, with a bias-corrected and accelerated (BCa) 95% confidence interval estimated using 5000 non-parametric bootstrap resamples.

Responder rates across follow-up time points were evaluated using Cochran’s Q test. Between-group comparisons according to GPE-defined responder status were performed using the independent-samples *t*-test for normally distributed continuous variables and the Mann–Whitney U test for non-normally distributed continuous variables. When multiple hypothesis tests were performed within a responder-analysis family, *p*-values were adjusted using the Benjamini–Hochberg false discovery rate (FDR) procedure. For responder proportions, 95% confidence intervals (CIs) were calculated using the Wilson score method. Spearman’s rank correlation analysis was used exploratorily to assess associations between baseline clinical variables and GPE-defined treatment response (GPE ≥ 6) at 1, 3, and 6 months. Because these correlation analyses were exploratory, raw, unadjusted *p*-values are reported and the full correlation matrix is provided in Supplementary Table S1. Statistical significance was set at *p* < 0.05 or adjusted *p* < 0.05 where a multiplicity correction was applied. All analyses were conducted using IBM SPSS Statistics version 27.0 (IBM Corp., Armonk, NY, USA). All 42 enrolled participants completed the prespecified follow-up assessments; therefore, no imputation for missing outcome data was required.

## 3. Results

### 3.1. Patient Flow and Baseline Characteristics

A total of 66 candidates were assessed for eligibility, of whom 42 met the inclusion criteria and were enrolled. Twenty-four patients were excluded: 13 did not meet clinical criteria, 2 declined to participate, and 9 were excluded during the pre-procedural safety assessment. The latter group included 5 patients with extensive vascularization and aberrant vessel courses overlying the procedural pathway, precluding a safe needle trajectory, and 4 with severe procedural anxiety. All 42 enrolled patients successfully completed the intervention protocol and the 6-month follow-up period.

The study cohort had a mean age of 44.4 ± 7.4 years (Table 1). The mean migraine history was 18.3 ± 8.8 years, and the population was highly treatment-resistant: 97.6% had failed prior greater occipital nerve (GON) blocks, 95.2% had failed GON pulsed radiofrequency, and 71.4% met the criteria for medication overuse at baseline (Table 2).

### 3.2. Primary and Secondary Longitudinal Outcomes

All longitudinal continuous outcomes differed significantly over time (Friedman tests, *p* < 0.001 for all parameters; Table 3). MHD decreased by a median of 17.0 days from baseline to Month 6 (BCa 95% CI: 13.0–19.0 days) based on 5000 bootstrap resamples.

Monthly Headache Days (MHD). The median monthly headache days (MHD) shifted from 29.0 at baseline to 6.0 at 6 months (Kendall’s W = 0.643). Post hoc pairwise comparisons confirmed these reductions were significant at all follow-up time points compared with baseline (*p* < 0.001).

Monthly Severe Headache Days and Attack Duration. Monthly severe headache days dropped from a median of 20.0 at baseline to 2.0 at 6 months (W = 0.768). Median attack duration also demonstrated a robust decline across the study period (W = 0.877).

Monthly Analgesic Use. The median number of days requiring analgesic use decreased from 15.0 days at baseline to 1.5 days at 6 months.

Pain Intensity. Mean pain intensity (VAS) decreased from 8.0 at baseline to 5.0 at all follow-up intervals (*p* < 0.001). Although statistically significant, the magnitude of improvement in pain intensity was less pronounced than the reduction in headache frequency.

**Figure 3:**
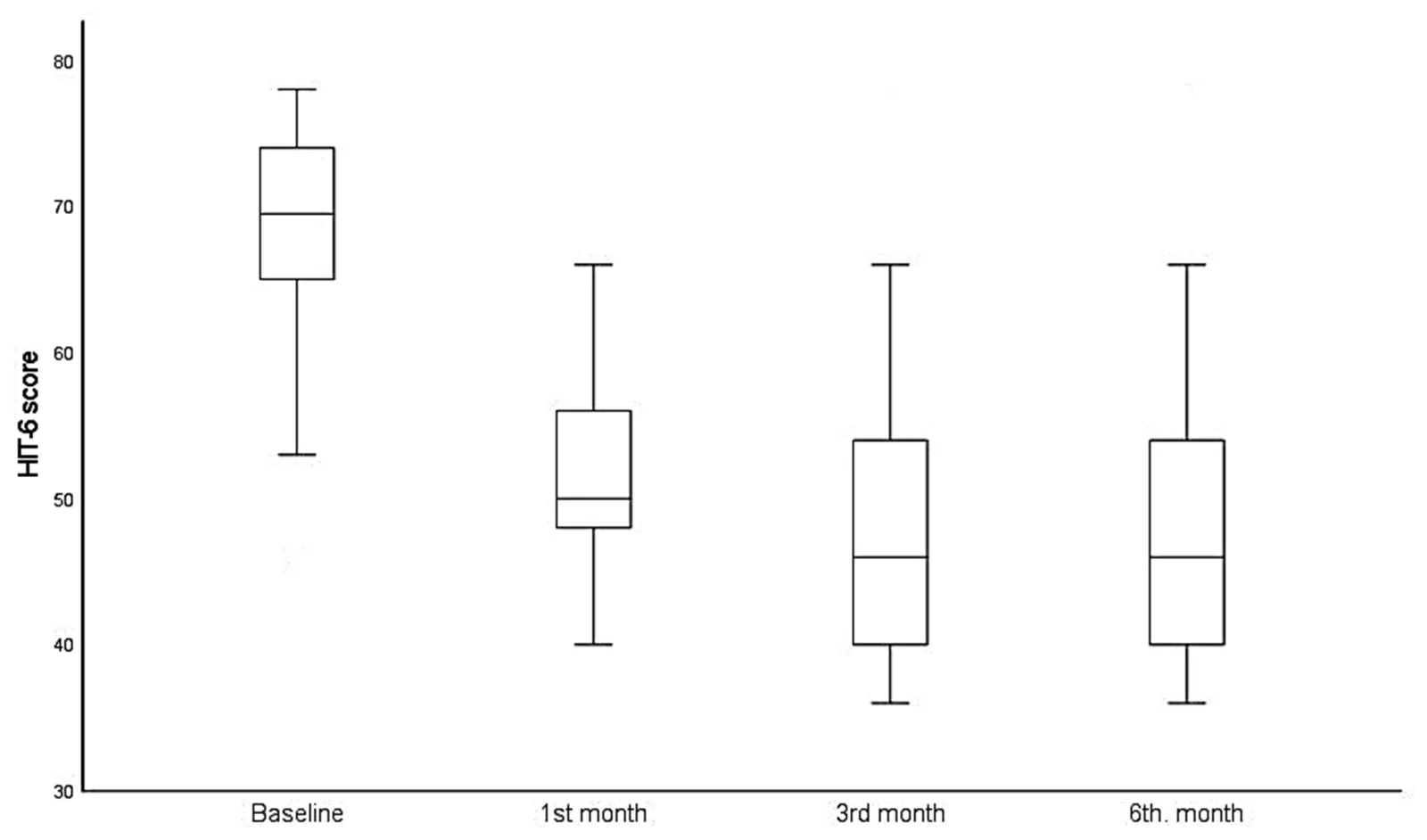
Distribution of Headache Impact Test (HIT-6) scores at baseline and at 1, 3, and 6 months after stellate ganglion block.

**Figure 4:**
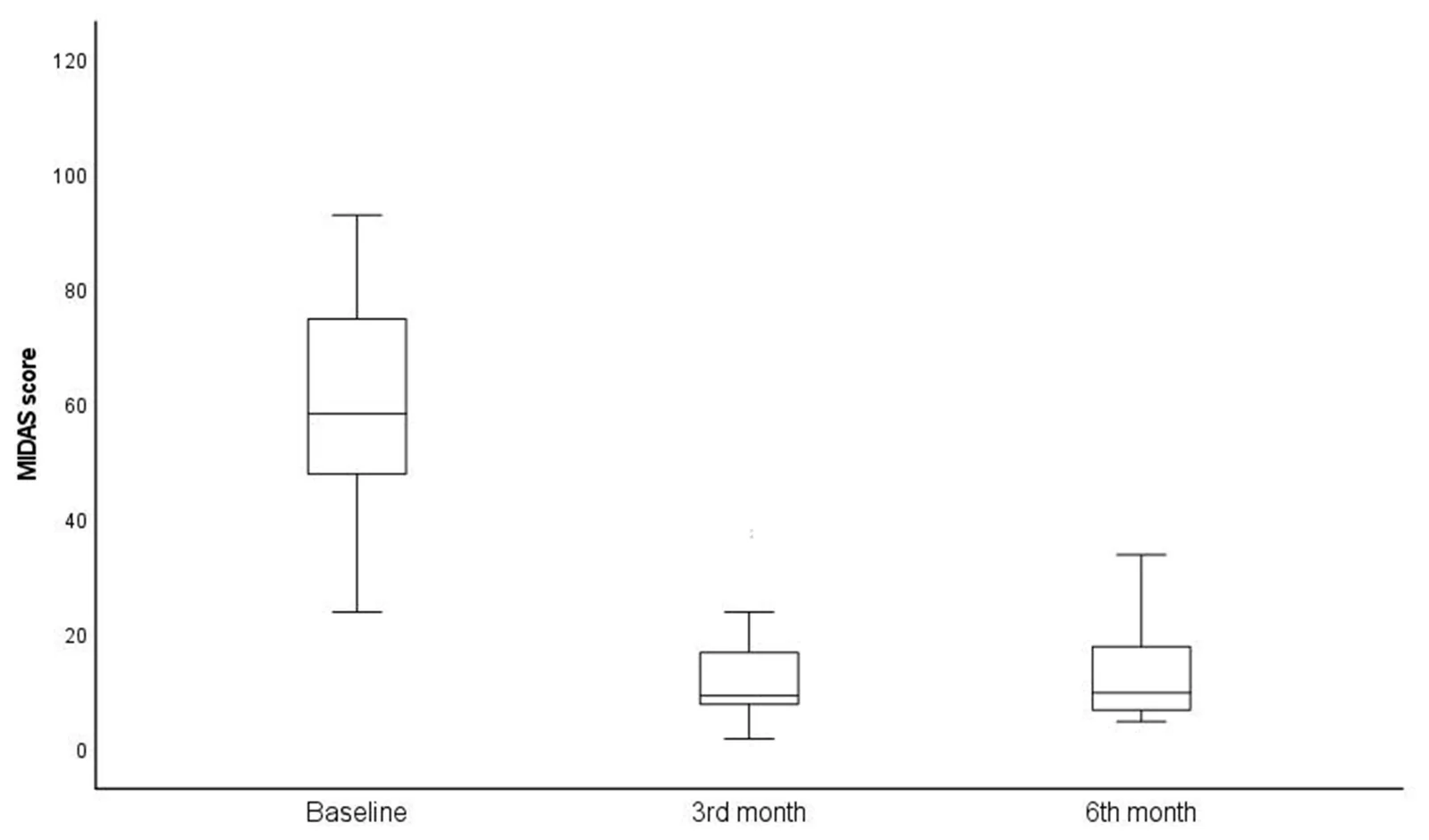
Distribution of Migraine Disability Assessment (MIDAS) scores at baseline and at 3 and 6 months after stellate ganglion block.

Disability Indices. Headache-related disability improved substantially; median HIT-6 scores decreased from 69.5 to 46.0 (W = 0.854), and MIDAS scores decreased from 58.5 to 10.0 by month 6 (Figure 3 and Figure 4).

### 3.3. Patient Satisfaction and Responder Rates

Clinically meaningful improvement (GPE ≥ 6) was reported by 83.3% of patients at month 1, 85.7% at month 3, and 76.2% at month 6. The proportion of GPE responders did not differ significantly across follow-up time points after FDR adjustment (Cochran’s Q, adjusted *p* = 0.074; Table 4).

**Table 4:** Clinically Meaningful Pain and Treatment Response Rates.

Outcome	Month 1, *n* (%) [95% CI]	Month 3, *n* (%) [95% CI]	Month 6, *n* (%) [95% CI]	Adjusted *p* *
Mean pain-intensity response (≥50% decrease in mean VAS)	8 (19.0) [10.0–33.3]	9 (21.4) [11.7–35.9]	6 (14.3) [6.7–27.8]	0.311
Mean pain-intensity response (≥30% decrease in mean VAS)	28 (66.7) [51.6–79.0]	29 (69.0) [54.0–80.9]	29 (69.0) [54.0–80.9]	0.717
MHD response (≥50% decrease in MHD)	37 (88.1) [75.0–94.8]	34 (81.0) [66.7–90.0]	31 (73.8) [58.9–84.7]	0.011
GPE response (GPE ≥ 6)	35 (83.3) [69.4–91.7]	36 (85.7) [72.2–93.3]	32 (76.2) [61.5–86.5]	0.074

Values are presented as *n* (%) with 95% confidence intervals calculated using the Wilson score method. Note: * Cochran’s Q test was used to compare responder proportions across the three follow-up time points. *p*-values for the responder analyses in this table were adjusted using the Benjamini–Hochberg false discovery rate (FDR) procedure. Adjusted *p* < 0.05 was considered statistically significant. Abbreviations: GPE, Global Perceived Effect; MHD, monthly headache days; VAS, Visual Analog Scale.

A ≥50% reduction in monthly headache days (MHD) was achieved by 88.1% of patients at month 1, 81.0% at month 3, and 73.8% at month 6; responder proportions differed significantly across follow-up time points after FDR adjustment (Cochran’s Q, adjusted *p* = 0.011; Table 4). At month 6, 73.8% of the cohort maintained a ≥50% reduction in MHD, whereas 14.3% achieved a ≥50% reduction in mean pain intensity (VAS), indicating a frequency-dominant response pattern.

### 3.4. Responder and Exploratory Correlation Analyses

At month 6, patients classified as responders (GPE ≥ 6; *n* = 32) had significantly better outcomes across all assessed clinical measures than non-responders (GPE < 6; *n* = 10) after FDR adjustment (all adjusted *p* < 0.001; Table 5). The median MHD was 5.5 days in responders versus 17.0 days in non-responders.

**Table 5:** Clinical Outcomes at 6-Month Follow-up in GPE Responders and Non-Responders.

Month 6 Outcome	GPE Responders (≥6), *n* = 32	GPE Non-Responders (<6), *n* = 10	Adjusted *p* *
Attack duration (hours)	6.0 (4.3–7.8)	12.0 (9.5–18.0)	<0.001
Mean pain intensity (VAS)	4.5 (4.0–5.0)	5.0 (5.0–6.3)	<0.001
Peak pain intensity (VAS)	6.0 (5.0–6.8)	7.0 (7.0–8.3)	<0.001
Monthly headache days (MHD)	5.5 (4.0–9.5)	17.0 (13.5–21.0)	<0.001
Monthly severe headache days	1.0 (1.0–3.0)	6.0 (4.0–16.8)	<0.001
Monthly analgesic use (days)	1.0 (1.0–3.0)	6.0 (4.0–9.0)	<0.001
HIT-6	42.0 (40.0–47.5)	56.0 (49.0–68.5)	<0.001
MIDAS	8.5 (7.0–14.0)	21.5 (10.8–42.0)	<0.001

Note: Values are presented as median (IQR; 25th–75th percentiles). * Mann–Whitney U tests; *p*-values were adjusted for multiple comparisons using the Benjamini–Hochberg false discovery rate (FDR) procedure. Adjusted *p* < 0.05 was considered statistically significant. Abbreviations: GPE, Global Perceived Effect; HIT-6, 6-item Headache Impact Test; MIDAS, Migraine Disability Assessment; VAS, Visual Analog Scale.

In the FDR-adjusted baseline responder analyses, baseline monthly headache days were lower among month 1 GPE responders than among month 1 non-responders (median: 27.0 vs. 30.0 days; adjusted *p* < 0.001). No other baseline demographic or clinical variable differed significantly between the responder and non-responder groups at months 1, 3, or 6 after FDR adjustment (Table 6).

**Table 6:** Baseline Demographic and Clinical Characteristics According to GPE-Defined Responder Status at Months 1, 3, and 6.

Variable	Month 1 Responders (*n* = 35)	Month 1 Non-Responders (*n* = 7)	Adjusted *p* *	Month 3 Responders (*n* = 36)	Month 3 Non-Responders (*n* = 6)	Adjusted *p* *	Month 6 Responders (*n* = 32)	Month 6 Non-Responders (*n* = 10)	Adjusted *p* *
Age, years	44.6 ± 7.8	43.1 ± 4.7	0.630 †	44.5 ± 7.7	43.5 ± 5.1	0.857 †	45.0 ± 7.7	42.3 ± 6.1	0.407 †
Duration since migraine diagnosis, years	18.0 (15.0–25.0)	10.0 (3.0–20.0)	0.294 ‡	17.0 (15.0–24.5)	13.5 (2.8–23.0)	0.336 ‡	19.2 ± 8.8	15.4 ± 8.8	0.407 †
Duration of chronic migraine, years	4.0 (2.0–10.0)	5.0 (2.0–7.0)	0.786 ‡	4.0 (2.0–9.8)	5.5 (1.8–7.8)	0.857 ‡	3.5 (2.0–5.8)	6.5 (2.8–10.0)	0.348 ‡
Mean attack duration, hours	24.0 (10.0–24.0)	24.0 (24.0–24.0)	0.294 ‡	24.0 (10.0–24.0)	24.0 (21.8–24.0)	0.458 ‡	24.0 (10.0–24.0)	24.0 (21.8–24.0)	0.407 ‡
Mean pain intensity, VAS	8.0 (7.0–8.0)	8.0 (7.0–8.0)	0.786 ‡	8.0 (7.0–8.0)	8.0 (6.8–8.3)	0.857 ‡	8.0 (7.0–8.0)	8.0 (7.0–8.0)	0.786 ‡
Peak pain intensity, VAS	10.0 (9.0–10.0)	10.0 (10.0–10.0)	0.294 ‡	10.0 (9.0–10.0)	10.0 (10.0–10.0)	0.315 ‡	10.0 (9.0–10.0)	10.0 (10.0–10.0)	0.132 ‡
Monthly headache days	27.0 (20.0–30.0)	30.0 (30.0–30.0)	<0.001 ‡	26.0 (20.0–30.0)	30.0 (30.0–30.0)	0.176 ‡	27.0 (20.0–30.0)	30.0 (20.0–30.0)	0.407 ‡
Monthly severe headache days	20.0 (16.0–25.0)	25.0 (20.0–30.0)	0.209 ‡	20.0 (16.0–25.0)	25.0 (22.3–30.0)	0.176 ‡	20.0 (16.0–25.0)	21.5 (20.0–26.3)	0.419 ‡
Monthly analgesic use, days	15.0 (8.0–16.0)	20.0 (15.0–20.0)	0.132 ‡	15.0 (8.5–19.0)	20.0 (15.0–22.5)	0.176 ‡	15.0 (8.5–20.0)	15.0 (13.8–20.0)	0.407 ‡
HIT-6	69.0 (65.0–73.0)	76.0 (66.0–76.0)	0.195 ‡	69.0 (65.0–73.8)	73.5 (65.8–76.5)	0.336 ‡	66.5 (63.0–72.8)	74.5 (69.8–76.0)	0.132 ‡
MIDAS	58.0 (47.0–75.0)	69.0 (61.0–75.0)	0.176 ‡	58.0 (47.0–75.0)	66.5 (58.5–79.5)	0.314 ‡	58.0 (47.0–75.0)	62.5 (54.0–75.0)	0.407 ‡

Note: Values are presented as mean ± standard deviation or median (interquartile range [IQR], 25th–75th percentiles), as appropriate. *p*-values were adjusted using the Benjamini–Hochberg false discovery rate (FDR) procedure within each follow-up time point. * Adjusted *p*-value; † independent-samples *t*-test; ‡ Mann–Whitney U test. Adjusted *p* < 0.05 was considered statistically significant. Abbreviations: GPE, Global Perceived Effect; HIT-6, 6-item Headache Impact Test; MIDAS, Migraine Disability Assessment; VAS, Visual Analog Scale.

Exploratory Spearman correlation analyses using raw, unadjusted *p*-values identified several negative associations between baseline clinical variables and GPE-defined treatment response. Higher baseline peak pain intensity was associated with lower response at month 6 (ρ = −0.352, *p* = 0.022), and higher baseline HIT-6 score was also associated with lower response at month 6 (ρ = −0.387, *p* = 0.011). Higher baseline monthly headache days were negatively associated with response at months 1 and 3 (ρ = −0.334, *p* = 0.030; ρ = −0.380, *p* = 0.013), higher baseline monthly severe headache days were negatively associated with response at month 3 (ρ = −0.316, *p* = 0.041), and higher baseline monthly analgesic-use days were negatively associated with response at months 1 and 3 (ρ = −0.363, *p* = 0.018; ρ = −0.318, *p* = 0.040). These exploratory correlation *p*-values were not adjusted for multiple testing; the full correlation matrix is provided in Supplementary Table S1.

### 3.5. Safety and Tolerability

Across the cohort, 210 protocol-defined scheduled SGB sessions were completed (42 participants × 5 sessions); scheduled bilateral sessions involved sequential blocks on both sides with a minimum of a 60-min interval. Transient Horner’s syndrome was observed in the majority of participants as an expected pharmacological effect of sympathetic blockade and was not classified as an adverse event. Six participants (14.3%) reported mild, self-limited adverse effects (headache, dizziness, shoulder discomfort, dyspnea, or injection-site pain/hematoma), all of which resolved spontaneously. No serious adverse events were documented. Adverse-event information was recorded primarily at the participant level rather than through prespecified procedure-level surveillance; therefore, the exact session timing and unilateral/bilateral attribution of individual events could not be reconstructed reliably.

## 4. Discussion

In this prospective, single-center, uncontrolled, single-group interventional study, ultrasound-guided stellate ganglion block (SGB) was associated with clinically meaningful improvements that were maintained for up to 6 months in patients with highly treatment-resistant chronic migraine. The most prominent finding was the marked reduction in monthly headache days (MHD), accompanied by substantial decreases in analgesic use and migraine-related disability. Parallel reductions in HIT-6 and MIDAS scores suggest that the observed decrease in headache frequency was accompanied by meaningful functional improvement in this highly treatment-resistant population.

The exploratory correlation analyses suggested that greater baseline headache burden, analgesic use, pain intensity, and headache-related impact may be associated with a lower patient-perceived response at selected follow-up points. Because these analyses were exploratory, involved multiple correlations, and used raw unadjusted *p*-values in a modest sample, they should not be interpreted as independent predictors of treatment response. Larger studies with prespecified multivariable models are needed to determine whether any of these baseline characteristics have reproducible prognostic value.

Chronic migraine remains a major cause of neurological disability worldwide [24,25]. Patients who fail multiple preventive therapies represent a particularly vulnerable subgroup with limited therapeutic options and high functional impairment [26]. While the European Headache Federation has emphasized the distinction between resistant and refractory migraine, our cohort reflects a particularly challenging clinical phenotype within the available therapeutic landscape [3]. Beyond pharmacological treatment failure, participants had a high baseline headache burden, frequent medication overuse, and extensive exposure to prior interventional treatments. In this context, the magnitude and persistence of the observed changes after SGB are clinically noteworthy, although the uncontrolled study design precludes causal attribution.

To maintain methodological transparency, the initial SGB was used to assess anatomical suitability and procedural tolerability rather than as a predictive diagnostic peripheral nerve block. No predefined quantitative response threshold was imposed for continuation. Although the protocol allowed participants who perceived no benefit and did not wish to proceed to discontinue after the initial SGB, none elected to do so; all 42 participants completed the four additional weekly sessions. For participants requiring bilateral SGB, blocks were performed sequentially with a minimum 60-min interval and continuous clinical monitoring. This approach avoided investigator-imposed enrichment of the cohort according to an early response threshold.

### 4.1. Potential Neuromodulatory Mechanisms

Several mechanisms may plausibly contribute to the clinical changes observed after SGB. Modulation of cervical sympathetic outflow could influence interactions between autonomic regulation and trigeminovascular nociceptive processing, with potential downstream effects on central sensitization [11,18,27]. The reduction in MHD observed in this cohort is compatible with a neuromodulatory effect, but the present clinical data cannot establish a specific mechanism or demonstrate a ‘neuromodulatory reset’. Such mechanistic interpretations should therefore be regarded as hypotheses for future physiological and imaging studies.

A possible mechanistic framework involves modulation of bidirectional signaling among trigeminovascular pathways, hypothalamic networks, and autonomic brainstem nuclei [7,8,28]. Anatomical studies also highlight the clinical relevance and variability of the cervicothoracic (stellate) ganglion [29]. Whether SGB alters a physiological ‘migraine threshold’ or directly modifies these networks remains uncertain. The present findings provide a clinical rationale for investigating these hypotheses using mechanistic study designs rather than evidence of a specific central mode of action.

### 4.2. Frequency Reduction Versus Pain-Intensity Reduction

These findings underscore the value of multidimensional outcome assessment—including headache frequency, pain intensity, and disability—when evaluating interventional treatments in highly treatment-resistant populations. A notable finding was the marked reduction in MHD despite a more modest change in pain intensity during remaining attacks. Specifically, 73.8% of patients achieved a ≥50% reduction in MHD, whereas 14.3% achieved a ≥50% reduction in mean VAS scores.

This dissociation suggests that the principal clinical signal in this cohort was a reduction in headache frequency rather than a comparable reduction in the intensity of remaining attacks. From a patient-centered perspective, fewer headache days may represent a meaningful improvement even when breakthrough attacks remain moderately painful. The concomitant decline in analgesic use may also be relevant in a population with a high prevalence of medication overuse [30].

Taken together, these findings suggest that SGB may have a role as an adjunctive interventional option for selected patients with highly treatment-resistant chronic migraine. The greater improvement in headache frequency than in pain intensity indicates that future trials should evaluate multidimensional endpoints rather than relying on pain-intensity measures alone. Whether this pattern reflects sympathetic neuromodulation, nonspecific treatment effects, or other mechanisms requires confirmation in controlled studies.

### 4.3. Interpretation in the Absence of a Control Group

A notable finding in our study was the marked reduction in median monthly headache days (MHD) from 29.0 at baseline to 6.0 at the 6-month follow-up. Because this was an uncontrolled cohort, the contribution of placebo effects, regression to the mean, natural fluctuation of migraine burden, and other nonspecific effects cannot be separated from the observed treatment-associated changes. The persistence of improvement across 6 months and the parallel reduction in monthly analgesic use support the clinical relevance of the observed signal, but causal efficacy cannot be established without a randomized controlled comparison.

### 4.4. Comparison with Existing Literature

Although the evidence base for stellate ganglion block (SGB) in chronic migraine remains limited, our findings are consistent with emerging observational data. Hou et al. reported improvements in migraine-related outcomes after SGB [19], and Yu et al. described reductions in headache frequency and attack duration in an elderly migraine cohort [20]. The present study extends this literature by focusing on a highly treatment-resistant population and by evaluating outcomes over 6 months.

Direct comparisons with established preventive therapies are not appropriate because of differences in study design, patient selection, treatment exposure, and the absence of a control group in the present study. Nevertheless, the clinical domains evaluated here—headache frequency, disability, and treatment response—are also central outcomes in trials of onabotulinumtoxinA and CGRP-pathway preventive therapies [31,32,33]. This overlap in outcome domains provides context for future controlled evaluation of SGB but should not be interpreted as evidence of equivalence or superiority. Rather than replacing established preventive therapies, SGB may warrant investigation as a potential adjunctive intervention or neuromodulatory bridge in selected highly treatment-resistant patients when conventional pharmacological and interventional options have provided insufficient benefit.

### 4.5. Safety and Procedural Considerations

The procedure was generally well tolerated in this cohort. Transient Horner’s syndrome was expected as a pharmacological sign of sympathetic blockade, and the documented adverse effects were mild and self-limited [34]. However, because adverse-event surveillance was not prespecified at the individual-procedure level, these findings should be interpreted as descriptive tolerability observations rather than as a comprehensive safety assessment.

Taken together, these tolerability observations support further controlled evaluation of SGB in appropriately selected patients. Its potential clinical role should be considered investigational and complementary to, rather than a replacement for, established preventive therapies.

### 4.6. Limitations

This study has several limitations. First, it was conducted at a single center with a modest sample size and no randomized or sham comparator. The absence of a control group precludes attribution of the observed changes specifically to SGB; placebo and contextual effects, regression to the mean, concurrent clinical care, and natural variability of chronic migraine may have contributed. The findings should therefore be interpreted as treatment-associated and hypothesis-generating. Second, multivariable predictive modeling was not performed, and the exploratory Spearman analyses involved multiple correlations with raw, unadjusted *p*-values; these findings should not be interpreted as independent predictors. Third, access to CGRP-targeted therapies was limited during the study period, so the cohort reflects a real-world therapeutic landscape in which newer preventive therapies were not routinely accessible.

Although participants were instructed to maintain prospective monthly headache records, completed forms were not available at every visit for every participant; some follow-up variables therefore relied on structured patient recall, introducing a potential risk of recall bias. In addition, adverse-event data were recorded primarily at the participant level rather than through prespecified procedure-level surveillance, limiting reliable attribution of individual events to a specific session or to unilateral versus bilateral treatment.

Finally, we acknowledge the evolving nomenclature in headache medicine. “Highly treatment-resistant” is used here as an operational descriptor of a tertiary pain-clinic population with substantial headache burden and extensive prior treatment exposure; it should not be interpreted as a separate formal EHF diagnostic category or as confirmation that all participants fulfilled every formal resistant/refractory criterion. Generalizability may therefore be greatest for similarly selected tertiary-care populations.

## 5. Conclusions

In this prospective, single-center, uncontrolled, single-group interventional study of patients with a highly treatment-resistant chronic migraine phenotype, ultrasound-guided SGB was associated with sustained 6-month reductions in headache frequency, analgesic use, and migraine-related disability. The greater reduction in MHD than in pain intensity suggests a frequency-dominant pattern of clinical improvement. Because the study lacked a randomized or sham comparator, these findings are preliminary, treatment-associated, and hypothesis-generating rather than evidence of intervention-specific efficacy. Within this context, SGB may represent a potential adjunctive intervention or neuromodulatory bridge for selected patients who remain symptomatic despite established preventive and interventional therapies. Confirmation in larger, multicenter randomized, preferably sham-controlled, trials is required.

## Figures and Tables

**Table 1:** Demographic Characteristics and Comorbidities.

Variable	Mean ± SD	*n* (%)
Age	44.4 ± 7.4	
Sex		
Female		29 (69.0)
Male		13 (31.0)
BMI	27.6 ± 5.0	
Marital status		
Married		38 (90.5)
Single		4 (9.5)
Educational status		
Primary school		13 (31.0)
Secondary school		7 (16.7)
High school		10 (23.8)
University		12 (28.6)
Comorbidities		
Hypertension		9 (21.4)
Thyroid dysfunction		9 (21.4)
Diabetes mellitus		3 (7.1)
Hyperlipidemia		3 (7.1)
Asthma/COPD		4 (9.5)
Fibromyalgia syndrome		10 (23.8)

Note: Continuous variables are presented as mean ± standard deviation (SD); categorical variables are presented as *n* (%). Abbreviations: BMI, body mass index; COPD, chronic obstructive pulmonary disease; DM, diabetes mellitus; FMS, fibromyalgia syndrome; HT, hypertension; HL, hyperlipidemia.

**Table 2:** Clinical Characteristics, Previous Treatments, and Stellate Ganglion Block Details.

Variable	Mean ± SD/Median (IQR)	*n* (%)
Time after migraine diagnosis (years)	18.3 ± 8.8	
Duration of chronic migraine (years)	4.0 (2.0–9.3)	
Pain location		
Right		6 (14.3)
Left		13 (31.0)
Side-shifting		11 (26.2)
Bilateral		12 (28.6)
Family history of migraine		20 (47.6)
Analgesic overuse		30 (71.4)
Prophylactic treatment *		
Beta-blockers		23 (54.8)
SNRI		21 (50.0)
Topiramate		14 (33.3)
TCA		10 (23.8)
Flunarizine		3 (7.1)
SSRI		2 (4.8)
Valproate		2 (4.8)
ACE inhibitors		1 (2.4)
History of BTX-A injection		12 (28.6)
Non-pharmacological therapies		
Acupuncture		14 (33.3)
Cupping		12 (28.6)
Leech therapy		3 (7.1)
Interventional pain treatment history *		
GON block		41 (97.6)
GON PRF		40 (95.2)
Sphenopalatine ganglion block		20 (47.6)
SPG pulsed radiofrequency		1 (2.4)
SON block		15 (35.7)
Trigger point injection		7 (16.7)
Side of the SGB		
Right		23 (54.8)
Left		6 (14.3)
Bilateral		13 (31.0)
Complications/side effects of SGB		6 (14.3)

Note: Continuous variables are presented as mean ± standard deviation (SD) or median (interquartile range [IQR]), as appropriate; categorical variables are presented as *n* (%). * Patients could have received more than one prophylactic or interventional treatment; therefore, percentages within these categories do not sum to 100%. Abbreviations: ACE, angiotensin-converting enzyme; BTX-A, botulinum toxin type A; GON, greater occipital nerve; PRF, pulsed radiofrequency; SGB, stellate ganglion block; SNRI, serotonin and norepinephrine reuptake inhibitor; SON, supraorbital nerve; SPG, sphenopalatine ganglion; SSRI, selective serotonin reuptake inhibitor; TCA, tricyclic antidepressant.

**Table 3:** Evaluation of Outcome Scales and Pain Scores After Stellate Ganglion Block.

Outcome	Baseline	Month 1	Month 3	Month 6	Friedman *p*	Kendall’s W
Attack duration (h)	24.0 (11.5–24.0)	6.5 (5.0–10.0)	6.5 (4.8–8.5)	6.5 (5.0–10.0)	<0.001	0.877
Mean pain intensity (VAS)	8.0 (7.0–8.0)	5.0 (4.0–5.0)	5.0 (4.0–5.0)	5.0 (4.0–5.0)	<0.001	0.932
Peak pain intensity (VAS)	10.0 (9.0–10.0)	6.0 (6.0–7.0)	6.0 (5.0–7.0)	6.0 (5.8–7.0)	<0.001	0.923
Monthly headache days (MHD)	29.0 (20.0–30.0)	9.0 (5.8–10.0)	7.5 (5.0–10.0)	6.0 (4.8–12.0)	<0.001	0.643
Monthly severe headache days	20.0 (16.0–25.0)	2.5 (1.0–5.0)	2.0 (1.0–4.3)	2.0 (1.0–5.0)	<0.001	0.768
Monthly analgesic use (days)	15.0 (10.0–20.0)	2.0 (1.0–4.0)	1.5 (1.0–4.0)	1.5 (1.0–4.0)	<0.001	0.708
HIT-6	69.5 (65.0–74.3)	50.0 (48.0–56.0)	46.0 (40.0–54.0)	46.0 (40.0–54.0)	<0.001	0.854
MIDAS	58.5 (47.8–75.0)	—	9.6 (8.0–17.0)	10.0 (7.0–18.0)	<0.001	0.804

Note: Values are presented as median (IQR; 25th–75th percentiles). Global longitudinal differences were assessed using the Friedman test, with Kendall’s W reported as the effect-size measure. Bonferroni-adjusted pairwise comparisons with baseline were *p* < 0.001 at each available follow-up time point for all outcomes. MIDAS was assessed at baseline and at months 3 and 6; therefore, no month-1 MIDAS value is shown. Abbreviations: HIT-6, 6-item Headache Impact Test; MIDAS, Migraine Disability Assessment; VAS, Visual Analog Scale.

## Data Availability

The data presented in this study are available from the corresponding author upon reasonable request.

## Supplementary Materials

The following supporting information can be downloaded at: https://www.mdpi.com/article/10.3390/medicina62091784/s1, Table S1. Exploratory Spearman rank correlations between baseline clinical variables and GPE-defined treatment response at months 1, 3, and 6.

## Abbreviations

CM	chronic migraine
SGB	stellate ganglion block
MHD	monthly headache days
VAS	Visual Analog Scale
HIT-6	6-item Headache Impact Test
MIDAS	Migraine Disability Assessment
GPE	Global Perceived Effect
GON	greater occipital nerve
PRF	pulsed radiofrequency
CGRP	calcitonin gene-related peptide
EHF	European Headache Federation
ICHD-3	International Classification of Headache Disorders, 3rd edition
LCM	longus colli muscle
CCA	common carotid artery
