# Supplementary material for: Clinical Outcomes of Ultrasound-Guided Stellate Ganglion Block in Highly Treatment-Resistant Chronic Migraine: A 6-Month Prospective Single-Group Interventional Study
Source: Medicina (Kaunas). 2026 Sep 16;62(9):1784. doi: 10.3390/medicina62091784 (PMC13609291; doi:10.3390/medicina62091784)
Supplement: Supplementary file 1 [file medicina-62-01784-s001.zip › medicina-4529916-supplementary.pdf]

Supplementary Table S1. Exploratory Spearman Rank Correlations Between Baseline Clinical Variables and GPE-Defined Treatment Response

Baseline variable	Month 1 response		Month 3 response		Month 6 response	
	**Spearman rho**	**p**	**Spearman rho**	**p**	**Spearman rho**	**p**
Age	0.116	0.464	0.082	0.608	0.196	0.213
Sex	0.023	0.885	−0.021	0.895	0.012	0.942
Duration of migraine diagnosis	0.233	0.138	0.166	0.292	0.123	0.438
Duration of chronic migraine	−0.053	0.737	−0.031	0.844	−0.238	0.129
Family history of migraine	0.043	0.789	−0.019	0.903	−0.139	0.381
Analgesic overuse	−0.283	0.070	−0.258	0.099	−0.230	0.143
Mean attack duration	−0.229	0.145	−0.196	0.213	−0.172	0.277
Mean pain intensity	0.034	0.831	−0.030	0.849	−0.060	0.708
Peak pain intensity	−0.282	0.071	−0.257	0.100	−0.352	0.022
Monthly headache days	−0.334	0.030	−0.380	0.013	−0.193	0.220
Monthly severe headache days	−0.281	0.071	−0.316	0.041	−0.145	0.359
Monthly analgesic-use days	−0.363	0.018	−0.318	0.040	−0.158	0.319
HIT-6	−0.289	0.064	−0.217	0.167	−0.387	0.011
MIDAS	−0.274	0.079	−0.230	0.142	−0.152	0.335

Note: Treatment response was defined as GPE ≥ 6. Values are Spearman rho and raw, unadjusted two-sided p-values. These analyses were exploratory and were not adjusted for multiple testing. GPE, Global Perceived Effect; HIT-6, 6-item Headache Impact Test; MIDAS, Migraine Disability Assessment.
